# Efficient Use of Information in Adaptive Management with an Application to Managing Recreation near Golden Eagle Nesting Sites

**DOI:** 10.1371/journal.pone.0102434

**Published:** 2014-08-06

**Authors:** Paul L. Fackler, Krishna Pacifici, Julien Martin, Carol McIntyre

**Affiliations:** 1 Agricultural and Resource Economics, North Carolina State University, Raleigh, North Carolina, United States of America; 2 Department of Applied Ecology, North Carolina State University, Raleigh, North Carolina, United States of America; 3 Patuxent Wildlife Research Center, United States Geological Survey, Laurel, Maryland, United States of America; 4 Florida Fish and Wildlife Conservation Commission, Fish and Wildlife Research Institute, St. Petersburg, Florida, United States of America; 5 National Park Service, Fairbanks, Alaska, United States of America; University of Lleida, Spain

## Abstract

It is generally the case that a significant degree of uncertainty exists concerning the behavior of ecological systems. Adaptive management has been developed to address such structural uncertainty, while recognizing that decisions must be made without full knowledge of how a system behaves. This paradigm attempts to use new information that develops during the course of management to learn how the system works. To date, however, adaptive management has used a very limited information set to characterize the learning that is possible. This paper uses an extension of the Partial Observable Markov Decision Process (POMDP) framework to expand the information set used to update belief in competing models. This feature can potentially increase the speed of learning through adaptive management, and lead to better management in the future. We apply this framework to a case study wherein interest lies in managing recreational restrictions around golden eagle (*Aquila chrysaetos*) nesting sites. The ultimate management objective is to maintain an abundant eagle population in Denali National Park while minimizing the regulatory burden on park visitors. In order to capture this objective, we developed a utility function that trades off expected breeding success with hiker access. Our work is relevant to the management of human activities in protected areas, but more generally demonstrates some of the benefits of POMDP in the context of adaptive management.

## Introduction

There is currently a paradigm shift in many conservation organizations. Increasingly, these organizations are asked to come up with clear conservation objectives, and to increase the efficiency in their use of conservation funds. In this context, science can help improve understanding about how a system will respond to potential management actions, and as a result help achieve conservation objectives more efficiently. Scientific experiments represent the most rigorous and efficient way to reduce structural (model) uncertainty, i.e., uncertainty about the behavior of a managed system. This observation sometimes leads to a recommendation to conduct adaptive management as a sequential, 2-step “learn then manage” process. Unfortunately, in many situations, experiments are not compatible with short term conservation objectives. For example, it may not be legally possible to implement an experiment that could potentially harm an endangered species, even if the learning could ultimately benefit the species and its habitat. Similarly, in the case of the control of disease or invasive species, policy makers may be reluctant to allow experiments that they view as risky. Furthermore, it is rarely optimal to postpone management in order to wait until the analyses of experiments are complete. These perspectives argue against this sequential “learn then manage” approach to adaptive management.

By contrast, a decision that takes into consideration scientific uncertainty but is focused simultaneously on reducing this uncertainty and on addressing both conservation and socio-economic objectives may be acceptable to management agencies. This is effectively the goal of adaptive management: identifying the best decisions given the current state of knowledge and specified management objectives, and recognizing that current learning will lead to future management gains. Indeed, structural uncertainty or model uncertainty has most often been addressed by conducting adaptive management [1]–[3], providing a decision framework to balance short term conservation objectives and learning to better manage a system in the future [4]. Adaptive management is a special case of structured decision making, which is a decision analytic framework that decomposes a problem into its components: management objectives; potential management actions; system models that project consequences of the potential actions on the system; an optimization method that uses these components to identify optimal decisions; and a monitoring program to provide estimates of the state of the system [4]. Outputs from the optimization process can generally be synthesized into a format that is easy to use by decision makers. For example, it is easy to create decision tables, which display the optimal decisions given information about the state of the system. The decision process that we have described also allows one to explore and understand the implications of choosing specific actions when one is uncertain about how the actions may influence the system (for example through simulations).

Adaptive management extends the basic Markov Decision Process (MDP) framework [5] by recognizing that the structure of the system that relates state and control variables to rewards and future states is generally not known with certainty. Essentially there is scientific uncertainty about how the system will respond to specific actions and thus the goal is to improve management by reducing this uncertainty. Specifically, adaptive management augments a model with a set of additional state variables that define a probability distribution over the unknown features of that structure. When the uncertainty is characterized by a discrete set of possible parameter values or fundamentally different model structures, this distribution can be represented by a discrete set of probability weights. As new information becomes available these beliefs can be updated using Bayes Rule [4].

In this paper we use a partially observable Markov Decision Process (POMDP) framework in order to extend the scope of the adaptive management paradigm. Traditionally, the POMDP approach deals strictly with the inability to directly observe the current state [6] and the resultant need to incorporate this uncertainty in computing expected rewards and changes in system state. Unfortunately this standard POMDP approach is not well suited for addressing the issue of structural uncertainty raised here. To overcome its limitations we have applied a POMDP approach (“extended POMDP”; Fackler and Pacifici [7]) that allows for both structural uncertainty and partial observability (i.e., the uncertainty about the state of the system due to imperfect monitoring) to be handled in a common framework; here we focus on structural uncertainty. To date most applications of adaptive management use only the realized values of the state variable, along with the transition probabilities, to update beliefs about model parameters. The use of the extended POMDP framework allows additional information signals to be used to update beliefs about system parameters. This can potentially increase the speed of learning, thus making learning more valuable.

In the remainder of the paper we briefly describe the extended POMDP approach (see Fackler and Pacifici [7], for more details and theory about this approach) and then highlight several features of this approach by modifying an existing case study wherein interest lies in managing recreational restrictions around golden eagle nesting sites [8]. The computer codes for our models are included in Supplement S1, and can be implemented with MDPSolve: (https://sites.google.com/site/mdpsolve/).

## Materials and Methods

### Addressing Structural Uncertainty with POMDPs

The standard MDP is defined by some state variables 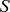, some possible actions 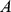, a reward function *R*(*S, A*) and a transition probability matrix that defines 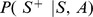 where 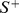 is the value of the state in the next period. In a standard MDP the goal is to find a decision rule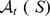 in order to maximize
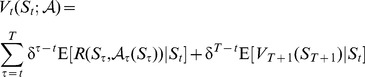
(1)where 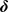 is a discount factor defined on [0,1], *t* is the current time at which a decision must be made (*t* = 1, …, *T*), 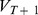 is a terminal reward function, and E[] is the expectation operator.

In the extended POMDP approach used here, the state variables are partitioned into those that are observable (*O*) and those that are unobservable (*U*): *S* = {*O*, *U*}. In addition an observed informational variable *Y*, called a signal, can be included that provides information about the unobserved state. The flexibility of this approach lies in defining a general dependency between the signal and current and/or future state variables. In some cases the information signal may be used to directly update (i.e., estimate) states, while in other cases it is simply a source of information that can increase the speed of learning (e.g., information used to differentiate competing structural models). For more information and examples of information signals, please see Fackler and Pacifici [7]. To make this variable sufficiently general we define the probability of the joint distribution of the next period’s state and signal conditional on this period’s state and action:
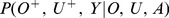
(2)


This framework extends the standard POMDP (for an overview of POMDPs see [9]) in two ways: first it decomposes the state into observable and unobservable components, thus incorporating the so-called Mixed Observability Markov Decision Process (MOMDP) introduced by [10], discussed further by [11], and applied to a conservation decision problem by [12]. Second, it uses the full joint distribution of future states and signals conditioned on current states and actions, allowing for more flexibility in defining dependence among observed and unobserved variables, which is an important feature for our ecological application.

The MOMDP approach replaces the unobservable variable *U* with a belief state variable *B*, representing a probability distribution over the values in *U*, which incorporates the history of the observable state, action and signal (note that *B* is a vector with non-negative elements that sum to 1). The belief state is updated after observation of *O* ^+^ and *Y* using Bayes Rule such that the probability for the future unobserved state, 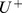 is given by:
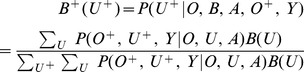
(3)


The goal is to find a decision rule 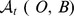 that is a function of the observable states and the belief states in order to maximize
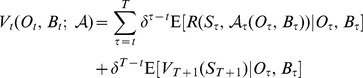
(4)


To address adaptive management problems, and thus structural uncertainty, in the extended POMDP framework, first define the unobservable state *U* to be the true model out of a set of *n_u_* possible models. Next define a probability distribution over *U*, *B*  =  *P*(*U*), and update *B* after observations of *O* ^+^ and *Y*. Note that traditional adaptive management only permits updating of model weights after observing the transition of the observable state variables (*O* ^+^) whereas here we can use observable state transitions (estimated from data or strictly observed) as well as signal information (*Y*), therefore potentially increasing the speed at which we can learn and discriminate between models of the system. We highlight the advantages of the extended POMDP approach below with an example based on [8].

### Recreational Restrictions near Golden Eagle Nesting Sites

Conflicts between human activities and wildlife survival are common [13] although the extent of the problem varies considerably across activities and species [14]–[17]. Recently, concerns have been raised as to the effect that recreational hikers may have on the breeding success of golden eagles in Denali National Park (Alaska, U.S.A.). The expansion of recreational hikers into pristine areas may disturb nesting golden eagles by increasing their energetic costs, changing their behavior and nest abandonment [18]. [8] developed a model to support the management of recreational restrictions around golden eagle nesting sites to learn about these impacts while managing them in an efficient manner. The management action consists of restricting access to hikers near known eagle nesting sites in order to reduce disturbance to breeding birds. In that study, management actions for a breeding season were determined prior to observing the occupancy status of the site in that season. Here we develop an alternative plan in which the management decision in season *t* is made after the occupancy status of the site in season *t* is known. The occupancy status at each site was determined by repeated visits at each site (up to three visits) prior to the start of the recreational hiking season. Although it is possible that biologists would overlook birds at a nest, [19] estimated that the average detection probability (i.e., the probability of detecting breeding golden eagles given that they are present at a site) was close to 1 (see Fig. 3 in [19]). Thus it is reasonable to assume that the occupancy status is known before the action is taken. As in [8] we focused on 25 potential sites that are accessible to hikers.

#### Single Site

Although the problem concerns the management of multiple sites it is useful to develop the model in terms of a single site and then to expand the problem to multiple sites. The state variable *S* is the occupancy status of the site which can be either empty or occupied (0 or 1). The management action *A* is whether hiking activity at an occupied site is either unrestricted or restricted (0 or 1). The state/action therefore takes on three possible values: (1) empty, unrestricted, (2) occupied, unrestricted and (3) occupied, restricted (we ignore the fourth case of a restricted empty site because it is not a logical action). The signal variable for this model concerns whether or not fledging is successful at the site. Table 1 specifies the possible events that can occur at each site. The columns of the table correspond to the current state and action (occupancy/restriction) and the rows correspond to the signal and future state (successful fledging/occupancy next year).

**Table 1:** Individual site joint state/signal probability matrix.

Signals and Future State			Current State/Action (*S*, *A*)		
*Y*_1_	*Y*_2_	*S* ^+^	occupied/restricted		
unrestricted/	restricted/	occupied			
successful	successful	next year	no/no	yes/no	yes/yes
no	no	no	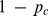	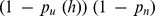	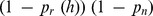
no	yes	no	0	0	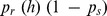
yes	no	no	0	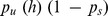	0
no	no	yes	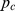	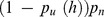	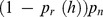
no	yes	yes	0	0	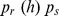
yes	no	yes	0	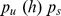	0

*p_c_* probability that an empty site is occupied (colonized) next year.

*p_n_* probability that a site is re-occupied next year if fledging is not successful.

*p_s_* probability that a site is re-occupied next year if fledging is successful.

*p_u_*(*h*) probability that fledging is successful if access to the site is unrestricted.

*p_r_*(*h*) probability that fledging is successful if access to the site is restricted.

*p_u_* and *p_r_* depend on the size of the arctic hare population *h*, which is assumed known at the time restriction decisions are made.

For reasons that will become clear when we move to a multiple site model, it is useful to split the signal into two variables, one for whether an occupied, unrestricted site was successful and the other for whether an occupied, restricted site was successful. The cells of Table 1 provide the joint probability distribution of the signals and next period state conditional on the current state and action. The success probabilities *p_u_* and *p_r_*, unrestricted and restricted respectively, depend on the level of the arctic hare population *h* (index of hare abundance, see [8]) which is assumed to be known at the time restriction decisions are made. Specifically



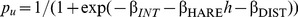
(5)and



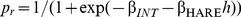
(6)Where 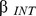 is the intercept, 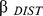 represents the impact of hiker related disturbance on the fledging success probability and 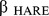 represents the impact of the level of the arctic hare population (for simplicity the hare level is set to its mean of 9.4; see Table 2 for parameter values).

**Table 2:** Parameter values of alternative models and assumptions.

Model	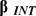	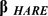	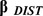	***p_u_***	***p_r_***
		*p_r_* certain			
1) no disturbance	−0.75	0.06	0	0.454	0.454
2) moderate disturbance	−0.75	0.06	−0.2	0.405	0.454
3) strong disturbance	−0.75	0.06	−0.6	0.313	0.454
		both *p_u_* and *p_r_* uncertain			
1) no disturbance	−0.85	0.06	0	0.429	0.429
2) moderate disturbance	−0.75	0.06	−0.2	0.405	0.454
3) strong disturbance	−0.65	0.06	−0.5	0.358	0.479
		*p_u_* certain			
1) no disturbance	−0.95	0.06	0	0.405	0.405
2) moderate disturbance	−0.75	0.06	−0.2	0.405	0.454
3) strong disturbance	−0.55	0.06	−0.4	0.405	0.504

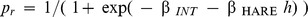

pc = 0.2315, pn = 0.9427, ps = 0.9573.

The hare level is treated as constant and set to its mean value (*h* = 9.4).

This is a case in which the joint distribution of the future state and signal, (*Y*, *S* ^+^) does not decompose in a way that would allow the use of standard POMDP (or even MOMDP). To see this, note that
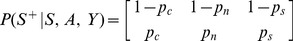
(7)where the columns represent (empty, not successful), (occupied, not successful) and (occupied, successful); *p_c_* is the probability that an empty site is occupied (colonized) next year, *p_n_* is the probability that a site is re-occupied next year if fledging is not successful, and *p_s_* is the probability that a site is re-occupied next year if fledging is successful. Notice that knowledge of *Y* makes knowledge of *A* uninformative for *S* ^+^, i.e. 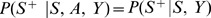. On the other hand the state transition matrix is
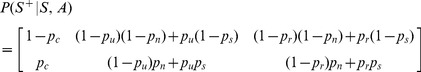
(8)(the dependence of *p_u_* and *p_r_* on the hare index is suppressed to reduce notational clutter).

As discussed in [20], given alternative conditional probability distributions *P*(*X|W*) and *P*(*X|Z*), a signal *W* is at least as informative as *Z* if there exists a probability matrix *T* such that
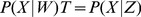
(9)


It can be seen from inspection that 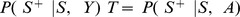 when
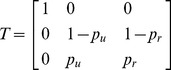
(10)


This formally verifies the intuitively reasonable result that knowledge of *Y* is more informative than knowledge of *A*.

#### Structural uncertainty

Structural uncertainty arises because there is considerable uncertainty about the effect of the restrictions on the fledging success probabilities (i.e., on *p_u_* and *p_r_*). In [8] it is assumed that *p_r_* is known but *p_u_* is uncertain. Three alternative models were suggested, one that represented a best estimate with *p_u_* <*p_r_*, one with no disturbance effect *p_u_*  =  *p_r_* and one with a strong disturbance effect *p_u_* <<*p_r_*. To model this, we augment the model with an additional state variable, taking on values of 1, 2 or 3, to represent which of these three models is, in fact, correct. This is a hidden or partially observed variable, and so we have two state variables, the occupancy status which is observed, and the disturbance effect, which is not. For this reason the mixed observability feature of the extended POMDP approach is useful, as this model could not be conveniently solved using standard POMDP approaches (see [12], for further discussion of this point).

The ability to learn arises from use of information about actual fledging success to update beliefs about the effect of restrictions. In a traditional adaptive management context, however, the updating of beliefs does not use *Y,* but instead relies solely on *S* ^+^ to update model belief weights. For this model the implication is that we must infer the success from the occupancy status next year to update our beliefs. Observing a high number of occupied sites next year is more likely if there was also a high number of successful fledgings. This is clearly a noisy signal, and it is to be expected that knowledge of fledging success is more useful than knowledge of next year's occupancy status.

Taken to the extreme if *p_n_*  =  *p_s_* (i.e., that fledging success this year does not influence the probability of occupancy next year) then the single site state transition would simplify, see Table 3.

**Table 3:** Single site state transition when if *p_n_*  =  *p_s_*.

future state	current state/action (*S*, *A*)		
*S* ^+^	*E*, *U*	*O*, *U*	*O*, *R*
empty	1−*p_c_*	1−*p_n_*	*1−p_n_*
occupied	*p_c_*	*p_n_*	*p_n_*

In this case *p_u_* and *p_r_* have completely dropped out of the transition probabilities and thus the occupancy history is uninformative about the effect of restrictions on fledging success. Although it was estimated that *p_s_* is slightly higher than *p_n_*, (see [8]) the effect is so small that traditional adaptive management (updating using only the occupancy data) would be expected to exhibit very slow learning.

What information is useful depends on what we take as known and what is treated as uncertain. In Martin et al. [8] the parameters were estimated under the assumption that *p_r_*, the success probability when nests are restricted and thus undisturbed, could be estimated (and thus, for simplicity, be treated as known with certainty) given the data available but that *p_u_* is not known. In this case the appropriate signal to reduce uncertainty is the number of unrestricted/successful sites (which implies that rows 1 and 2 and rows 4 and 5 of Table 1 can be added together). To explore other alternatives, suppose instead that in our historical record the nesting territories were disturbed by hikers. In this case *p_u_* is considered known (because unrestricted sites are potentially disturbed by hikers) but there is uncertainty about *p_r_* and the number of nesting territories that are restricted and successful is the appropriate signal (implying that rows 1 and 3 and rows 4 and 6 of Table 1 can be added together). More generally, however, the historical record might reflect a mix of disturbed and undisturbed sites in which case both *p_r_* and *p_u_* are uncertain and both the number of restricted/successful sites and the number of unrestricted/successful sites would be useful in making estimates more precise.

Under the assumption (as in [8]) that *p_r_*, the success probability when nests are restricted and thus undisturbed, was estimated given the data available (and thus considered known) the uncertainty centers around the value of β*_DIST_* (eqn 5). As in [8] three alternative models are considered representing (1) no disturbance effect (β*_DIST_* = 0) (2) a moderate disturbance effect (β*_DIST_*  = −0.2), which is the model considered most likely in [8] and (3) a strong disturbance effect (β*_DIST_*  = −0.6). In addition, two alternative assumptions are examined. One alternative is that *p_u_* is known but there is uncertainty about *p_r_*. A constant value of *p_u_* results when β*_INT_* + β*_DIST_* is constant. Setting *p_u_* equal to the value in the moderate disturbance case of 0.405 results in a value of β*_INT_* + β*_DIST_*  = −0.95. The intermediate assumption is that there is uncertainty about both 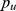 and *p_r_*. Parameter values for this case are set to represent a model that is half way between the two extreme cases.

Table 2 provides parameter values for the three alternative assumptions and the three alternative models (evaluated at the mean hare level). Note that when there is uncertainty about 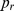 the no disturbance case gives the “best” outcomes because the unrestricted probability is lower for the other models and the restricted probability is constant. On the other hand when *p_u_* is uncertain the high disturbance model gives the best outcomes because it provides the greatest response to restricting access. This is seen in the values of *p_u_* and *p_r_* (evaluated at the mean hare level) in Table 2.

#### Multiple sites

The discussion thus far has focused on the transition probabilities for a single site. For a model with *N* sites to manage the state variable, *S*, is the number of occupied sites and the action, *A*, is the number of occupied sites that are restricted. Furthermore there are two possible signal variables, the number of unrestricted/successful sites, *Y*_1_, and the number of restricted/successful sites, *Y*_2_.

When there are multiple sites there may be multiple ways to arrive at the same (*Y*, *S* ^+^) pair. To illustrate how this arises consider a case with a single signal for the number of occupied but unrestricted sites that have successful fledging. For example, suppose that there are *N* = 2 sites; Table 4 lists the possible outcomes of assigning the 2 sites to the four possible outcome categories along with the values of the *Y* and *S* ^+^. The tabled values represent the number of sites in each category with the number of unrestricted and successful sites, *Y*, equal to the sum of columns 2 and 4 and next period's number of occupied sites, *S* ^+^, equal to the sum of columns 3 and 4 and. Rows 5 and 6 both result in the same signal/future state combination (1,1). In row 5, however, it occurs because there is one site that, though successful, is not re-occupied the following year and the other site is not successful but is occupied in the following year. In row 6 there is a unsuccessful site that is not re-occupied and a successful site that is re-occupied.

**Table 4:** Enumeration of States and Signals with 2 Sites.

NE	SE	NO	SO	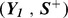
2	0	0	0	0,0
1	0	1	0	0,1
0	0	2	0	0,2
1	1	0	0	1,0
0	1	1	0	1,1
1	0	0	1	1,1
0	0	1	1	1,2
0	2	0	0	2,0
0	1	0	1	2,1
0	0	0	2	2,2

The tabled values represent the number of sites in each category. The number of unrestricted and successful sites, *Y*_1_, is the sum of columns 2 and 4 (SE and SO). The number of occupied sites next period, *S* ^+^, is the sum of columns 3 and 4 (NO and SO).

NE: either restricted or not successful/empty next period.

SE: unrestricted, successful/empty next period.

NO: either restricted or not successful/occupied next period.

SO: unrestricted, successful/occupied next period.

Table 5, using the moderate disturbance case parameters (Table 2) and the category count approach discussed in [21], displays the probability matrix for the *N* = 2 case. To obtain the probability matrix associated with the (*Y*, *S* ^+^) pairs any rows associated with a specific (*Y*, *S* ^+^) pair must be added together. In this case rows 5 and 6 must be summed to obtain the probability of getting 1 occupied site and having 1 successful unrestricted site.

**Table 5:** Joint state/signal probability matrix for 1 and 2 sites.

signal, future state	state/action (*S*, *A*)					
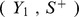	0,0	1,0	1,1	2,0	2,1	2,2
*N* = 1						
0,0	0.769	0.034	0.051			
0,1	0.231	0.561	0.949			
1,0	0	0.017	0			
1,1	0	0.388	0			
*N* = 2						
0,0	0.591	0.026	0.039	0.001	0.002	0.003
0,1	0.356	0.439	0.741	0.038	0.061	0.096
0,2	0.054	0.130	0.220	0.315	0.533	0.901
1,0	0	0.013	0	0.001	0.001	0
1,1	0	0.004	0	0.019	0.016	0
1,1	0	0.298	0	0.026	0.020	0
1,2	0	0.090	0	0.435	0.368	0
2,0	0	0	0	0.000	0	0
2,1	0	0	0	0.013	0	0
2,2	0	0	0	0.150	0	0

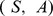
 = (i, j): i sites are occupied & j sites are restricted 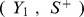 = (i, j): i unrestricted sites have successful fledging & j sites are occupied next year.

#### Utility function

In addition to exploring alternative parameter values for underlying models, the utility function used here differs from that of [8]. Previously, the objective was to maximize hiker access subject to a penalty if a target expected success rate was not met. Here we use the reward function
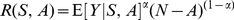
(11)With *N* defined as the number of sites, *A* the number of occupied sites that are restricted, and α = 0.85 (here *Y* is interpreted as the number of successful sites). This utility function trades off between expected breeding success 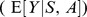 and hiker access (*N - A*) in a smooth way and rewards more of both. Also a discount factor of δ = 0.98 is used. Solving the undiscounted problem may overvalue learning as any increase in knowledge is amortized over an infinite future [3]. The use of a small but positive discount rate discourages such over-probing.

## Results

**Figure 1:**
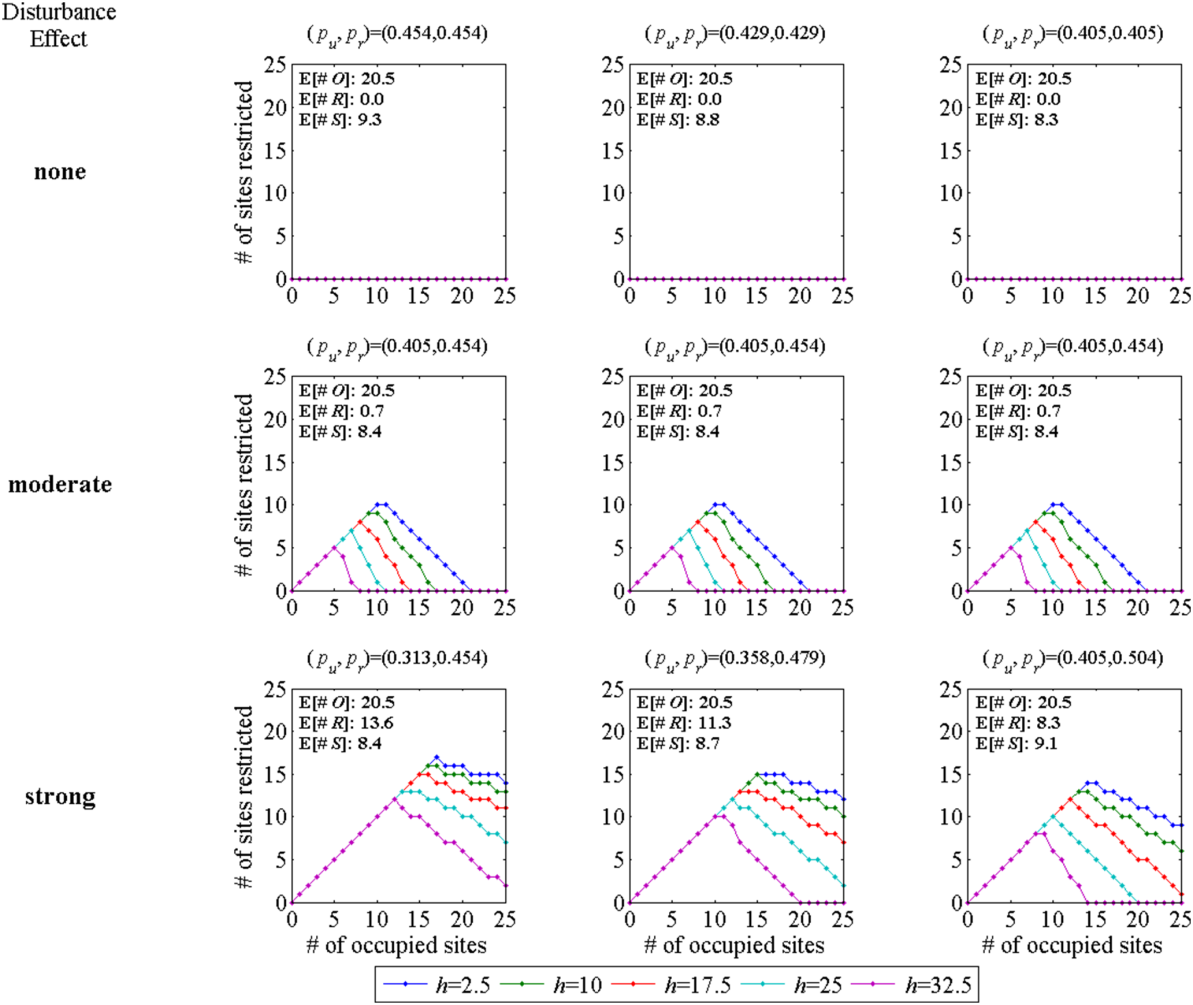
Optimal number of restricted sites with no structural uncertainty. The values of *p_u_*, *p_r_* are evaluated at the average hare level *h* = 9.4. The colored lines indicate the optimal number of sites to be restricted sites (# of sites restricted: from 0 to 25 sites) for 5 levels of index of hare abundance (from 2.5 to 32.5). Each subplot also shows the long-run expected number of occupied (E[#*O*]), restricted (E[#*R*]) and successful (E[#*S*]) sites. Note that the number of restricted sites can never be greater than the number of occupied sites.

Optimal rules for the number of restricted sites when there is no structural uncertainty are shown in Figure 1. In this figure (and all subsequent ones) the columns represent the cases of *p_r_* known, both *p_u_* and *p_r_* uncertain and *p_u_* known (see section: *Recreational Restrictions Near Golden Eagle Nesting Sites, Structural Uncertainty*). The rows in Figure 1 correspond to the cases of no disturbance effect, moderate effect and strong effect. Note that the middle row repesents the base case; these three plots are purposely identical to make it easier to make comparisons among alternatives.

The subplots in the top row, associated with models with no disturbance effect, show the intuitively reasonable result that no restrictions are ever called for. For the other two rows in which there is some disturbance effect, it is optimal to restrict all occupied sites up to a maximum that depends on the hare level (note that only occupied sites are potentially restricted so the restriction curves must lie on or below the 45 degree line). More hares lead to a greater success probability and hence less need for restrictions to achieve a given level of success. For the strong disturbance effect (the bottom row) the number of restrictions decreases as we move from the *p_r_* known (left column) to the *p_u_* known (right column) case because the increasing success probabilities again leads to a lower need for restrictions to achieve a given expected success level.

**Figure 2:**
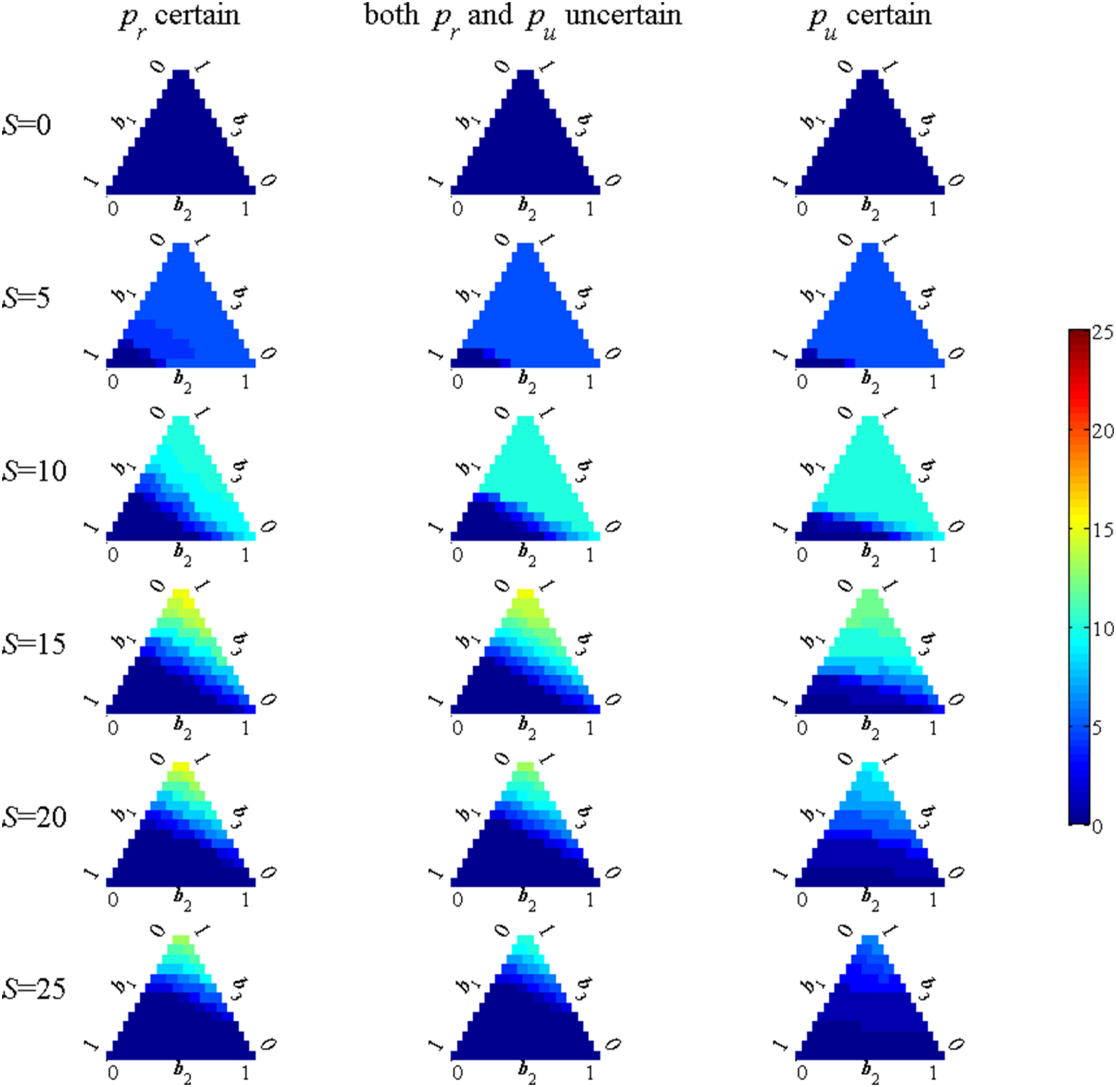
Number of restricted sites with active learning using information optimally. Each row of subplots corresponds to a specified number of occupied sites (*S*). The subplots show the optimal action for each belief value, with the three corners representing complete certainty in one of the models (lower left for the no disturbance model, lower right for the moderate disturbance model and top for the strong disturbance model). The number of possible restrictions ranges from 0 to *S* (the number of restricted sites can never be greater than the number of occupied sites).

When there is structural uncertainty the optimal decision rule depends not only on the number of occupied sites and the hare level but also on the degrees of belief in the three models. To simplify the presentation, however, the problem is solved treating the hare level as a constant set at its mean level. The complete decision rules are shown in Figure 2, with columns again representing the three alternative assumptions concerning which parameters are uncertain. The rows of subplots correspond to alternative numbers of occupied sites (*S*) with each subplot showing a ternary plot that displays the optimal strategy as a function of the belief weights. The lower left ([1 0 0]) corner corresponds to complete certainty in the no-disturbance model where no restrictions are ever optimal. The lower right ([0 1 0]) corner corresponds to complete certainty in the moderate disturbance effect case. The upper ([0 0 1]) corner corresponds to the complete certainty in the strong disturbance effect case. The colors in the plots represent the optimal number of restrictions, ranging form 0 to 25. It should be noted again that the number of restricted sites can never be greater than the number of occupied sites. Hence, by definition, the plots for *S* = 0 in the top rows of subplots are all blue.

**Figure 3:**
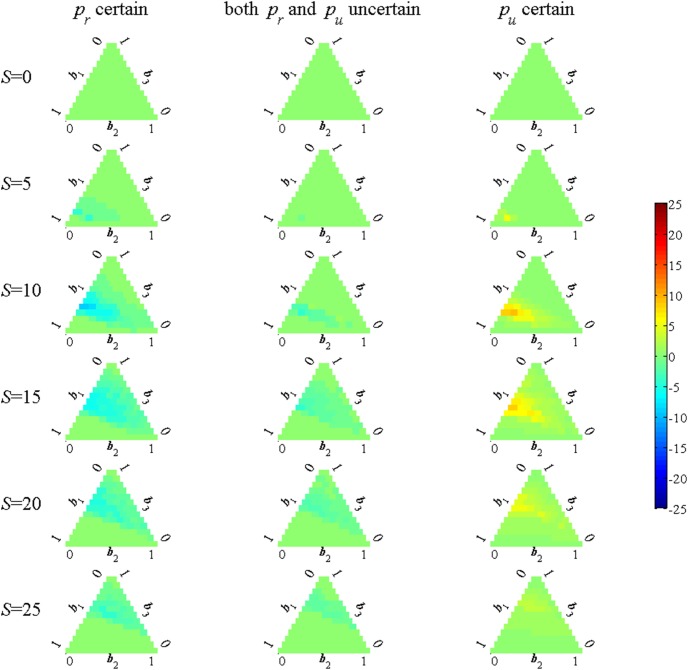
Probing activity using information optimally. Differences in the number of restrictions between the active management strategy (shown in Figure 2) and the passive management strategy. Green values indicate no probing activity, blue values represent fewer restrictions with the active strategy, yellow/red values indicate more restrictions with the active strategy.

A common distinction in the adaptive management literature concerns the distinction between active and passive learning strategies [4]. Passive learning strategies use decision rules derived under the assumption that the current beliefs will not change in the future. The difference in the strategies is therefore a measure of the degree to which it is optimal to engage in probing behavior in order to learn. The number of restrictions by which the active management strategy, shown in Figure 2, differs from that of the passive strategy is shown in Figure 3. The number of restrictions used in an active strategy is higher than for the passive strategy (yellow/red cells) in the *p_u_* known case. This reflects the fact that restricted sites lead to learning about *p_r_* but not *p_u_*. Similarly, in the case that *p_r_* is known it is optimal to use fewer restrictions in the active case (blue cells) because unrestricted sites lead to learning about *p_u_*. In the intermediate case with uncertainty about both *p_u_* and *p_r_* whether there is probing activity and the kind of activity depends on the current belief weights. The comparison reveals the intuitively reasonable result that more restrictions are imposed when the main uncertainty is about the effect of restrictions and fewer are imposed when the main uncertainty is about the effect of disturbance.

The strategy that uses the success information contrasts strongly with a strategy based on a traditional adaptive management approach, which only updates beliefs based on the realized value of the state variable after the state transition occurs but does not allow beliefs to be updated using any additional information. When only the occupancy status is used to update beliefs there is almost no difference between the number of restrictions used in the active and passive cases (there are a few specific belief values with 1 or 2 site differences between the active and passive strategies). The informational content of the future occupancy status about success rates is very slight and stems entirely from the small difference in the re-occupation probability if the previous season had a successful fledging or not.

**Figure 4:**
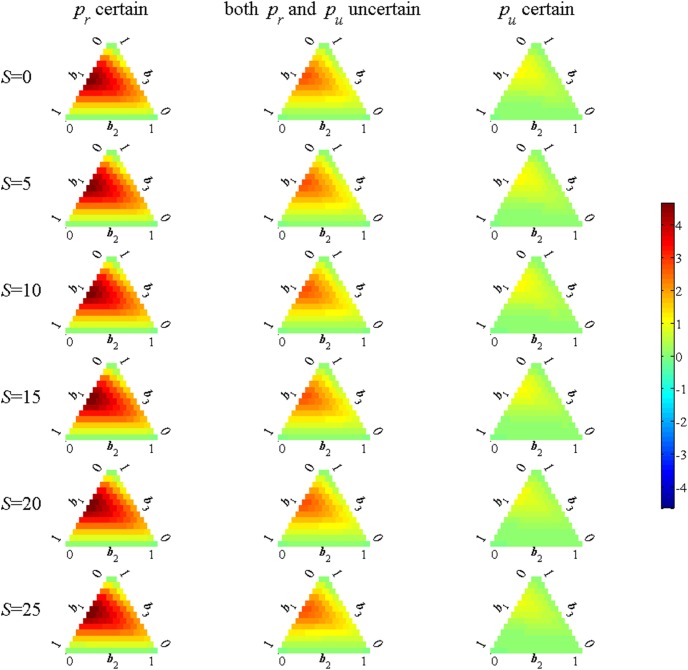
Comparison of value results from use of signal. Each subplot gives the percent increase in value using the best signal relative to the no signal case. Green values indicate equal value, blue values represent decrease in value with the signal, yellow/red values indicate an increase in value with the signal.

Figure 4 shows the percent increase in value due to the additional use of the success information, which can result in over 4% increase in value. The level of the value function is somewhat difficult to interpret (it is the sum of discounted utility values), so a scaleless relative increase in value is used. It should be pointed out, however, that the largest gains in value occur in a region of the belief space that it not very likely, namely where the moderate effect model has low weight and there is a more or less even split between the no and strong effects models (near the left side of the ternary plot); such a bimodel belief is deemed to be unlikely.

**Figure 5:**
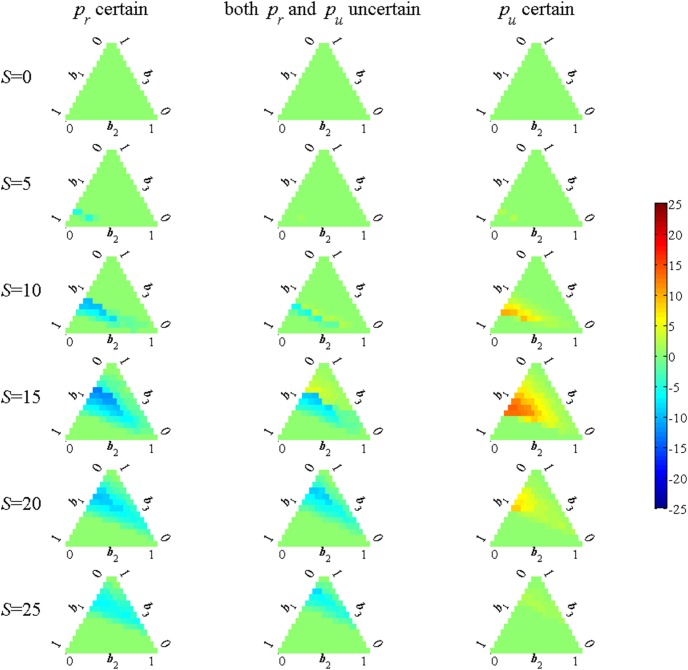
Probing activity with the signal equal to the number of successes. Green values indicate no probing activity, blue values represent fewer restrictions with the adaptive strategy, yellow/red values indicate more restrictions with the active strategy.

It is important to note that different information is used in the three alternative cases. When *p_r_* is certain the information variable is the number of unrestricted sites that are successful, whereas in the *p_u_* known case it is the number of restricted sites that are successful. The intermediate case with both parameters uncertain uses both of these information variables. These signals are arguably the best use of the success data to update model weights. Suppose instead that only the number of successes is known but not whether they are associated with restricted or unrestricted sites. The strategy used with this less informative signal is shown in Figure 5. Comparing Figures 2 and 5 shows that more restrictions are used when *p_u_* is known and more sites are unrestricted when *p_r_* is known. When only the number of successes is known to learn effectively it is best to either restrict all the occupied sites or none of them as then one knows what the signal implies for the efficacy of the action taken. This means that far more probing is required to obtain a similar learning outcome which could make a difference as to whether a strategy is accepted by managers. The strategy is relatively effective, however, in that the difference in value relative using the optimal signal is quite slight (less than half a percent).

**Figure 6:**
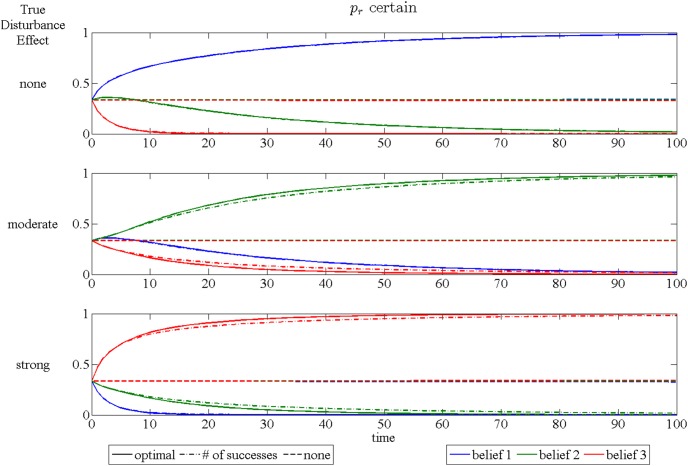
Time paths of expected beliefs when *p_r_* is known. The three subplots represent cases for each of the three possible disturbance effect models. Each plot shows time paths of expected beliefs over 100 years. Line color is associated with the belief in a specific disturbance effect: blue – no disturbance, green –moderate disturbance, red – strong disturbance. Line type represents the signal used for updating beliefs: solid – full information, dash/dot – number of successes only, dashed – no signal.

**Figure 7:**
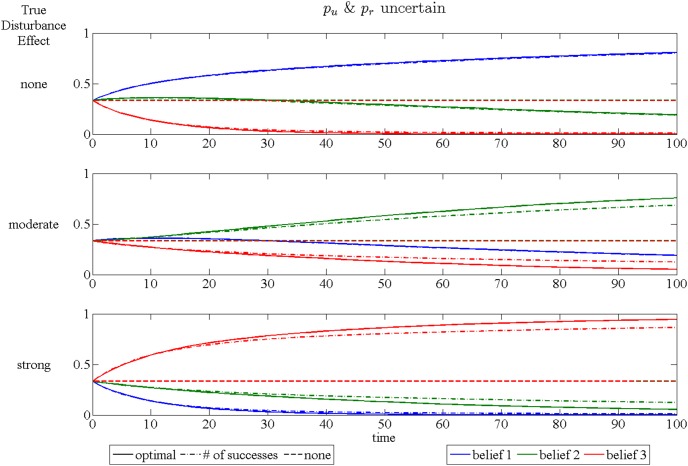
Time paths of expected beliefs when both *p_u_* and *p_r_* are uncertain. The three subplots represent cases for each of the three possible disturbance effect models. Each plot shows time paths of expected beliefs over 100 years. Line color is associated with the belief in a specific disturbance effect: blue – no disturbance, green –moderate disturbance, red – strong disturbance. Line type represents the signal used for updating beliefs: solid – full information, dash/dot – number of successes only, dashed – no signal.

**Figure 8:**
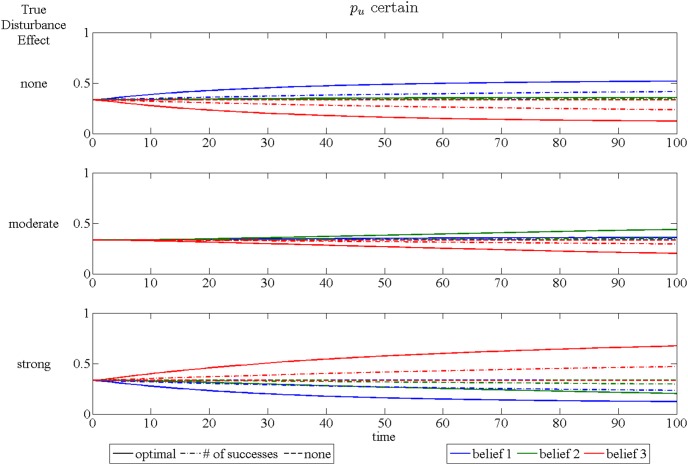
Time paths of expected beliefs when *p_u_* is known. The three subplots represent cases for each of the three possible disturbance effect models. Each plot shows time paths of expected beliefs over 100 years. Line color is associated with the belief in a specific disturbance effect: blue – no disturbance, green –moderate disturbance, red – strong disturbance. Line type represents the signal used for updating beliefs: solid – full information, dash/dot – number of successes only, dashed – no signal.

The speed at which learning occurs can be examined using plots of the expected time paths of the belief weights. These are shown for the three different assumptions in Figures 6, 7 and 8. Each figure has three subplots representing which of the three models is, in fact, correct. These plots are obtained by simulating 10000 time paths and averaging for each year over a 100 year period, with the initial beliefs putting equal weight on each model and the initial occupancy of 20 out of 25 sites.

The expected speed of learning is faster when the curve for the true model approaches 1 more quickly. The main message of these plots is that learning occurs at a faster pace using information optimally (shown as the solid lines). If only the number of sucesses is used without distinguishing whether the successful sites are restricted (dash/dot lines), the speed of learning is slower, although how much slower depends on both the true model of the disturbance effect and the assumption made concerning the uncertainty of *p_r_* and *p_u_*. The traditional adaptive management approach, which uses only the state variable to update beliefs, is totally ineffective at producing any learning (dashed lines). This is due to the fact that success only raises the probability of future occupancy by a small amount (*p_n_* and *p_s_* are nearly equal) and hence occupancy is only slightly helpful in learning about the effect of restrictions on success.

## Discussion

The extended POMDP framework is a flexible approach for making decisions under uncertainty that is applicable to a wide range of conservation and management problems (Fackler and Pacifici [7]). It builds on the approach of [12] and extends it by adding modeling flexibility in how observable variables relate to state variables. The extended POMDP framework provides a way to incorporate both structural and observational uncertainty, though only the former is explored here. The approach allows both observed state variables and other observable variables (like fledging success) to be used in updating beliefs about model parameters. By utilizing more information than is contained in the state variables alone, the approach has the ability to expedite learning and to improve our ability to more quickly discriminate among competing models of system dynamics. Furthermore the approach used here allows the observation (signal) variables to be conditioned on both the current and future state variables, thereby permitting great flexibility in modeling complicated systems.

We demonstrate the use of the framework for addressing structural uncertainty using a case study involving the potential need to restrict recreational hiking near golden eagle nesting sites [8]. In this example it is evident that the information content of the signal makes a large difference in determining both the optimal strategy and the value of the strategy. It is also clear that the extent of probing activity to undertake and under what situations learning takes place depends on where the greatest uncertainty exists (*p_u_*, *p_r_*, or both). The active adaptive management case always takes probing actions to learn about the parameter with the greatest uncertainty. This contrasts in two ways with the adaptive management approach implemented in [8]. First, previously only a passive adaptive strategy was used in which no probing occurs. Second, only the state transitions were used to update model weights. Both of these factors lead to a slower rate of learning. In this case the rate of learning would be especially slow because the state transitions (future occupancy status) provided very little information about the effects of restrictions on fledging success. Not surprisingly, the value of the traditional approach is significantly less than one which uses an informative signal.

The extended POMDP approach provides the opportunity to explore the use of multiple information signals. By identifying and isolating where the greatest uncertainty exists, the extended POMDP approach has the potential to identify which signal can provide the greatest reduction in uncertainty. The choice of information signal may ultimately depend on additional factors (e.g., cost of obtaining different information signals), but the flexibility to identify and choose the most valuable signal could add substantial value to a management program.

We also believe that the extended POMDP approach can be useful in exploring other features of adaptive management and structural uncertainty. Traditionally the alternative models apply to the transition model for the state variables, but they could also apply to other outcome variables that enter the reward (utility) function. This latter possibility has not been allowed in previous adaptive management applications (see [4]) in which only the realization of the state variables is used to update beliefs. In principle one could expand the state space to include these additional outcome variables, as was done in [8]. Doing this, however, increases the dimensionality of the problem, increasing computational problems, and causes interpretive difficulties because the action does not depend on these variables. In the extended POMDP approach uncertainty in the reward can be handled seamlessly in an intuitive fashion.

It is worth noting that there are several critical distinctions between the work done here and the original modeling approach used in [8]. Most notably we have assumed that the occupancy status (year *t*) and reproductive success (year *t*-1) are known before the decision for year *t* is made. This occupancy information is usually known around May of a particular year, but one assumption of the previous model was that the decision regarding which sites to restrict to hikers needed to be made the previous winter. Our new approach allowed us to relax this assumption, and we are now able to use information about occupancy in May right before the hiking season that starts in June. We therefore developed a new set of optimal actions assuming that we could obtain the relevant information before making a decision. This in turn allowed us to be far more selective in restricting sites and hence could achieve better outcomes and faster learning.

Our ecological application of the extended POMDP approach demonstrates the potential of this recently developed decision analytical framework to address natural resource management issues. Another interesting feature of our application is that we integrated the extended POMDP framework with a category count model [21]. One benefit of using a category count model is that this class of models is well suited to deal with spatially structured populations, which is a common characteristic of many managed ecological systems (e.g., metapopulations). Category count models also naturally account for discrete stochastic processes that are analogous to demographic stochasticity or genetic drift but in an occupancy context. For instance, in a metapopulation context patch extinction and colonization are stochastic processes applied to discrete units. In this example, the colonization in period *t* of a patch that is not occupied in period *t*-1 can be viewed as a Bernoulli trial with a given probability. Similar to demographic stochasticity and genetic drift, the importance of this source of variation to modeling is the size of the population of interest, which in the case of patch occupancy corresponds to the number of patches occupied. Ignoring this source of uncertainty when implementing adaptive management may lead to overestimating the rate of learning. Finally, we have demonstrated that the use of a carefully chosen signal can significantly increase the efficiency of learning and therefore improve management through faster learning about the ecological system. We hope that this new decision analytical framework will be useful to solve other important management and conservation problems.

## Supporting Information

Supplement S1 (ZIP)
